# A Potential High-Risk Clone of *Pseudomonas aeruginosa* ST463

**DOI:** 10.3389/fmicb.2021.670202

**Published:** 2021-05-28

**Authors:** Yanyan Hu, Wenjing Peng, Yifan Wu, Hui Li, Qi Wang, Huahua Yi, Rong Zhang, Bing Shao, Kui Zhu

**Affiliations:** ^1^Clinical Microbiology Laboratory, The Second Affiliated Hospital of Zhejiang University, School of Medicine, Zhejiang University, Hangzhou, China; ^2^National Center for Veterinary Drug Safety Evaluation, College of Veterinary Medicine, China Agricultural University, Beijing, China; ^3^Beijing Key Laboratory of Diagnostic and Traceability Technologies for Food Poisoning, Beijing Center for Disease Prevention and Control, Beijing, China; ^4^Department of Respiratory and Critical Care Medicine, Ruijin Hospital, Shanghai Jiao Tong University School of Medicine, Shanghai, China

**Keywords:** hypervirulence, multi-drug resistance, pyocyanin, ST463, *Pseudomonas aeruginosa*

## Abstract

*Pseudomonas aeruginosa* is one of the most common opportunistic pathogens, which causes severe nosocomial infections because of its well-known multidrug-resistance and hypervirulence. It is critical to curate routinely the epidemic *P. aeruginosa* clones encountered in the clinic. The aim of the present study was to investigate the connection between virulence factors and antimicrobial resistance profiles in epidemic clones. Herein, we found that ST463 (O4), ST1212 (O11), and ST244 (O5) were prevalent in 30 isolates derived from non-cystic fibrosis patients, based on multilocus sequence type (MLST) and serotype analysis. All isolates were multidrug-resistant (MDR) and each was resistance to at least three classes of antibiotics in antimicrobial susceptibility tests, which was consistent with the presence of the abundant resistance genes, such as *bla*_OXA–50_, *bla*_PAO_, *aph*(3′), *catB*7, *fosA*, *crpP*, and *bla*_KPC–2_. Notably, all *bla*_KPC–2_ genes were located between IS*Kpn6*-like and IS*Kpn8*-like mobile genetic elements. In addition, classical exotoxins encoded by *exoU*, *exoS*, and *pldA* were present in 43.44% (13/40), 83.33% (25/30), and 70% (21/30) of the isolates, respectively. The expression of *phz* operons encoding the typical toxin, pyocyanin, was observed in 60% of isolates (18/30) and was quantified using triple quadrupole liquid chromatograph mass (LC/MS) assays. Interestingly, compared with other MLST types, all ST463 isolates harbored *exoU*, *exoS* and *pldA*, and produced pyocyanin ranging from 0.2 to 3.2 μg/mL. Finally, we evaluated the potential toxicity of these isolates using hemolysis tests and *Galleria mellonella* larvae infection models. The results showed that ST463 isolates were more virulent than other isolates. In conclusion, pyocyanin-producing ST463 *P. aeruginosa*, carrying diverse virulence genes, is a potential high-risk clone.

## Introduction

*Pseudomonas aeruginosa* is one of the most common gram-negative pathogens and is associated with ubiquitously acute and chronic infections, especially cystic fibrosis (Ji et al., 2013). The worldwide spread of *P. aeruginosa* poses a threat to global public health (Tacconelli et al., 2018). *P. aeruginosa* exhibits various mechanisms of antimicrobial resistance, including the use of efflux pumps, biofilm formation, an impermeable outer membrane, an adaptable genome, antibiotic-inactivating enzymes, mobile resistance genes, and target mutations (Curran et al., 2018; Horcajada et al., 2019; Zhu et al., 2019). Recently, the increasing incidence of multidrug resistance (MDR), particularly for carbapenems, has induced a new crisis involving nosocomial *P. aeruginosa* infections (Curran et al., 2018; Horna et al., 2019).

Various virulence factors have been demonstrated to contribute to *P. aeruginosa* infection. For example, type III effectors (exotoxins ExoS, ExoT, ExoY, and ExoU), type VI effectors (PldA), adherence factors (type IV pili, flagella), alginate, elastase, and biosurfactant rhamnolipid (Karatuna and Yagci, 2010; Boulant et al., 2018; Luo et al., 2019) play crucial roles in mortality (Juan et al., 2017). It should be noted that ExoU-positive *P. aeruginosa* is more likely to be resistant to multiple antibiotics, such as carbapenems, cephalosporins, fluoroquinolones, and aminoglycosides (Hu et al., 2017), which further exacerbates infections and increases mortality (Horna et al., 2019). Interestingly, *exoU* has been reported to be mutually exclusive with *exoS*, a common gene in *P. aeruginosa* (Vareechon et al., 2017). Nevertheless, the coexistence of *exoS* and *exoU* enhances antibiotic resistance in *P. aeruginosa* (Horna et al., 2019). Moreover, pyocyanin, belonging to the family of phenazines, is the key virulence factor in *P. aeruginosa*. Pyocyanin is synthesized from chorismic acid through a series of biosynthetic enzymes encoded by the *phz* gene cluster (Supplementary Figure 1). Previous studies showed that pyocyanin can not only promote the pathogenicity to host cells by disrupting electron transport, cellular respiration, and energy metabolism (Rada and Leto, 2013), but also modulates bacterial physiology, such as survival, iron acquisition, biofilm formation, and antibiotic tolerance (Chincholkar and Thomashow, 2014; Zhu et al., 2019).

Recently, numerous epidemic *P. aeruginosa* strains have been described worldwide. For instance, ST175, ST235, and ST111 are high-risk clones with MDR profiles, among which ST235 is highly associated with *exoU* (Cholley et al., 2014). Infections caused by such strains often have a worse prognosis than infections with other strains. The combination of MDR and virulence factors always restricts the implementation of therapeutic options, thus there is an urgent need to investigate resistance and virulence characteristics to combat *P. aeruginosa* infections. The misuse and overuse of antibiotics, serving as a dominant driving force of resistance, might further shape the evolutionary trajectory of *P. aeruginosa* in the clinic and the environment. To date, the correlations between the presences of virulence factors, antibiotic resistance, and the genotype of *P. aeruginosa* in non-cystic fibrosis patients remain unclear. The present work investigated and characterized epidemic clones in a non-outbreak situation to shed light on the treatment options for *P. aeruginosa*-associated infections.

## Materials and Methods

### Bacterial Isolation

Thirty *P. aeruginosa* isolates were collected from 30 non-CF patients from the Second Affiliated Hospital of Zhejiang University School of Medicine from 2009 to 2018. The Second Affiliated Hospital of Zhejiang University School of Medicine is a general hospital with 3,200 beds, in which carbapenem-resistant *P. aeruginosa* (CRPA) had reached to 38.9% according to recent hospital surveillance. Thirty CRPA strains were randomly chosen from our previously sequenced genomes based on the sample source, isolation time, virulence factor, sequence type (ST), and carbapenemase genes. Specifically, the isolates were collected from sputum (*n* = 13), CVC (central vascular catheter, *n* = 3), blood (*n* = 3), urine (*n* = 3), feces (*n* = 3), pus (*n* = 4), and one sample with an unknown source. Detailed clinical information is shown in Supplementary Table 1. Before the experiments, all the isolates were re-identified using Matrix-assisted laser desorption/ionization-time of flight mass spectrometry (Bruker Daltonics, Billerica, MA, United States).

### Antimicrobial Susceptibility Testing

All isolates were tested with 16 kinds of antimicrobials, including aminoglycosides (amikacin, gentamicin, and tobramycin), β-lactam combination agents (ceftazidime-avibactam, cefoperazone-sulbactam, and piperacillin-tazobactam), cephems (ceftazidime, cefepime), monobactam (aztreonam), carbapenems (imipenem, meropenem), polymyxin (colistin, polymyxin B), and fluoroquinolones (ciprofloxacin, levofloxacin, and lomefloxacin). The minimum inhibitory concentrations (MICs) of the isolates were determined using the classic micro-broth dilution method following the operations in the Clinical and Laboratory Standards Institute’s performance standards (CLSI M100-S29) (Wayne, 2019). *P. aeruginosa* strain ATCC 27853 was chosen as a standard control for the antimicrobial susceptibility tests.

### Extraction of Pyocyanin

*P. aeruginosa* isolates were cultured on Luria-Bertani (LB) agar plates for 12 h, after which a single colony was selected for culture in LB broth at 37°C with 200 rpm shaking for 16 h. After centrifugation 13,000 × *g*, the supernatant was collected, extracted twice with chloroform (5:3 v/v), and vortexed. The chloroform phase was kept after centrifugation (5,000 × *g*, 10 min) and mixed with 0.2 M HCl (3:1 v/v). The red phase was collected after centrifugation (5,000 × *g*, 10 min), extracted with one-third the volume of chloroform containing NaHCO_3_, and the chloroform phase (blue) was collected (El-Zawawy and Ali, 2016). The extract was dissolved with 90% acetonitrile for high performance liquid chromatography (HPLC)-mass spectrometry (MS) detection. The HPLC-MS apparatus (Shimadzu, HPLC/MS-8045, Kyoto, Japan) was equipped with a Shim-pack GIST-HP C18 column (2.1 mm × 50 mm, 3 μm, Shimadzu) at an oven temperature of 35°C and a flow-rate of 0.3 mL/min. The gradient program was applied with the mobile phase consisting of solvent A (0.1% formic acid in acetonitrile) and solvent B (0.1% formic acid in water) as follows: 95–70% of B for 0–5.00 min, 70–50% of B for 5.00–5.10 min, 50–30% of B for 5.10–7.10 min, 30–0% of B for 7.10–11.10 min, held at 0% B for 11.10–13.00 min, 0–95% of B for 13.00–14.00 min, and maintained at 95% B for 14.00–16.00 min. The positive electrospray ionization (ESI+) mode was chosen to analyze pyocyanin. The MS parameters for pyocyanin are shown in Supplementary Table 2. The MS acquisition parameters used were as follows: gas temperature, 300°C; drying gas, 10 L/min; heating Gas, 10 L/min; DL Temperature, 250°C; heat block temperature, 400°C; second pole collision gas, argon gas; and CID gas volt, 17 kPa.

### Toxicity Evaluation

*P. aeruginosa* isolates were incubated in brain heart infusion (BHI) agar containing 5% sheep blood for hemolytic experiments. Virulence genes were analyzed by using BLAST software (SRST2 Toolkit version 0.2.0; Inouye et al., 2014), and the database of virulence genes at the NCBI. The virulence of *P. aeruginosa* isolates was evaluated *in vivo* using the *Galleria mellonella* larval infection model, and eight strains (1615, 1802, E211-2, 1608, 1617, 1104, ZR16, and 1109) were selected to analyze their characteristics. Strains 1617 (ST1212), 1104 (ST244), ZR16 (ST463), and 1109 (ST463) are pyocyanin-producing isolates, and 1615 (ST1076), 1608 (ST1212), 1802 (ST1212), and E211-2 (ST274) are pyocyanin-negative isolates. PA14 was used as a reference strain for pyocyanin expression, and its mutant ΔPA (*phz* genes cluster deleted) (Dietrich et al., 2013) was used as a negative control to evaluate the contribution of pyocyanin to the pathogenicity of *P. aeruginosa*. To prepare the inoculum, bacteria were grown for 12 h at 37°C with 200 rpm shaking, washed in sterile phosphate-buffered saline (PBS) after centrifugation at 3,000 × *g*, and then adjusted to a final concentration of 10^5^ colony forming units (CFU)/mL using a Nephelometer (Merieux, Nürtingen, Germany). A 10 μL aliquot of suspended strains (10^3^ CFU of bacteria) was injected into each larva and incubated at 37°C. Larvae were considered as dead if they did not respond to touch. The survival rates of *G. mellonella* larvae were recorded. The statistical analysis in this study is performed using GraphPad Prism 8 (GraphPad Inc., La Jolla, CA, United States). Continuous variables were described using the mean ± SD and categorical variables as the number (percentage). *T*-tests were conducted to assess the normal distribution of continuous data, while the Chi-squared or Fisher’s exact test were used to assess the categorical data. A *P*-value < 0.05 was considered statistically significant.

### DNA Extraction and Genetic Analysis

Genomic DNA of all 30 isolates was extracted using a Wizard genomic DNA purification kit (Promega, Beijing, China) according to the manufacturer’s instructions. The genomic DNA was then sequenced using the Illumina HiSeq X10 platform (Illumina, San Diego, CA, United States) with the 150-bp paired-end strategy. Raw reads were trimmed and assembled to contigs using SPAdes version 3.11.1 (Bankevich et al., 2012). Assembled contigs were analyzed *via* the Center for Genomic Epidemiology website to screen for the presence of acquired antimicrobial resistance genes (ARGs)^1^ (Boolchandani et al., 2019). The multilocus sequence type (MLST)^2^ (Larsen et al., 2012) and serotype^3^ (Thrane et al., 2016) were also determined. Virulence-associated genes and mobile genetic elements (MGEs) were collected from the NCBI database and were identified using SRST2 Toolkit version 0.2.0. The genomes of the 27 pyocyanin-producing isolates were obtained from the *Pseudomonas* Genome Database^4^. The phylogenetic tree was analyzed using Parsnp in the Harvest package based on the core genome sequences of the eight *P. aeruginosa* ST463 strains (Schürch et al., 2018). The tree was then visualized using the online tool iTOL (Cui et al., 2020).

## Results

### Clinical Characteristics of *P. aeruginosa* Isolates

MLST and serotype analysis revealed that all the isolates belonged to 14 MLST types: ST463 (8/30), ST244 (4/30), ST1212 (4/30), ST1076 (3/30), ST274 (2/30), ST769 (1/30), ST782 (1/30), ST3080 (1/30), ST235 (1/30), ST836 (1/30), ST260 (1/30), ST2438 (1/30), ST494 (1/30), and ST508 (1/30); and six serotypes: O5 (5/30), O6 (4/30), O10 (1/30), O11 (8/30), O3 (4/30), and O4 (8/30). The most prevalent ST type was ST463 (8/30), followed by ST1212 (4/30) and ST244 (4/30). Consistent with previous observations (Parkins et al., 2018; Horcajada et al., 2019), *P. aeruginosa* ST463 strains were associated with serotype O4, while ST244 was associated with serotype O5. The connections between serotypes and STs in other isolates are displayed in Supplementary Table 1. These data indicated that the sequence diversity of *P. aeruginosa* clones was high among the patients.

### Antimicrobial Resistance

**FIGURE 1:**
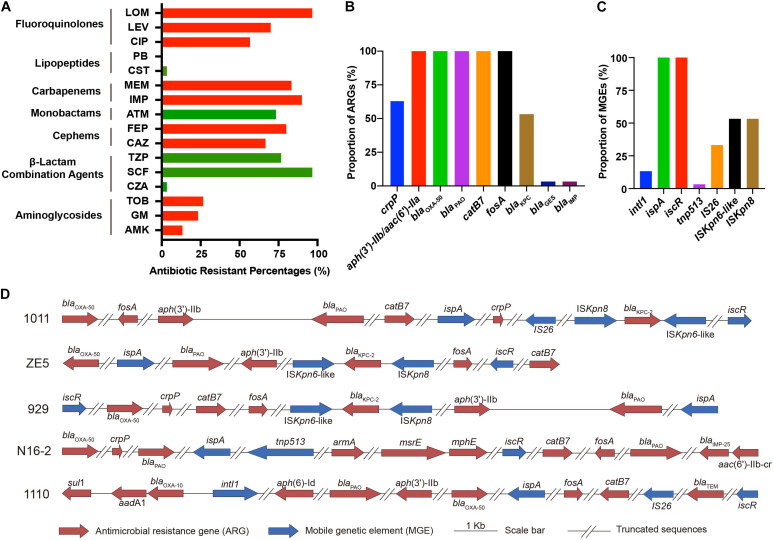
Phenotypical and genetic profiles of antimicrobial resistance in *P. aeruginosa* isolates. **(A)** Antibiotic resistance profiles. Proportion of antimicrobial resistance genes (ARGs) **(B)** and mobile genetic elements (MGEs) **(C)** in the isolates. **(D)** Schematic diagram of representative ARGs and MGEs in five isolates. The *bla*_KPC–2_ genes in *P. aeruginosa* 1011, ZE5, and 929 are surrounded by IS*Kpn6*-like, IS*Kpn8*, and other MGEs (*ispA*, *iscR*). *P. aeruginosa* N16-2 harbors MGEs *ispA*, *tnp513*; and *P. aeruginosa* 1110 harbors *intl1*, IS*26*, *iscR*. All strains share similar patterns.

Among all *P. aeruginosa* isolates, the antimicrobial resistance rates of ceftazidime-avibactam, cefoperazone-sulbactam, piperacillin-tazobactam, ceftazidime, cefepime, aztreonam, imipenem, meropenem, ciprofloxacin, levofloxacin, and lomefloxacin were 3.33% (1/30), 96.67% (29/30), 76.67% (23/30), 66.67% (20/30), 80% (24/30), 73.33% (22/30), 90% (27/30), 86.67% (26/30), 56.67% (17/30), 70% (21/30), and 96.67% (29/30), respectively (Table 1 and Figure 1A). Generally, there was a high proportion of resistance against the combinations of β-lactams because of the presence of intrinsic resistant genes *bla*_OXA–50_ and *bla*_PAO_. Remarkably, carbapenem-resistance genes (*bla*_KPC–2_, *bla*_GES–1_, and *bla*_IMP–9_) were identified in 60% of the isolates (18/30, comprising 16 *bla*_KPC–2_, 1 *bla*_GES–1_, and 1 *bla*_IMP–9_) (Figure 1B). In all 16 strains, *bla*_KPC_ was flanked by IS*Kpn6*-like and IS*Kpn8* mobile genetic elements (MGEs). In addition, aminoglycoside-resistance genes [*aph*(3′) or *aac*(3)-IId, *aac*(6′)-IIb] were present in all isolates. The fluoroquinolone-resistance gene (*crpP*) was harbored by 63.33% of the isolates (19/30). In addition, some MGEs, such as *intl*1, *ispA*, *iscR*, *tnp513*, and IS*26* were associated with diverse ARGs (Figures 1C,D).

**TABLE 1:** Antibiotic susceptibility profiles of *P. aeruginosa*.

Antimicrobial agents	MIC range (μg/mL)	MIC_50_ (μg/mL)	MIC_90_ (μg/mL)	Susceptible %	Intermediate %	Resistant %
AMK	0.5– > 64	2	>64	80.00	3.33	16.67
GM	1– > 64	2	>64	73.33	0	26.67
TOB	≤0.25– > 128	≤0.25	>128	73.33	0	26.67
CZA	≤1/4–128/4	4/4	8/4	96.67	0	3.33
SCF	8/4– > 256/4	256/4	>256/4	0	3.33	96.67
TZP	≤8/4– > 256/4	256/4	>256/4	13.33	10	76.67
CAZ	≤4–256	64	128	26.67	6,67	66.67
FEP	≤4– > 256	256	>256	10	10	80
ATM	≤8– > 256	256	>256	13.33	13.33	73.33
IMP	4– > 256	128	>256	0	10	90
MEM	2– > 256	128	>256	10.00	3.33	86.67
CST	1–4	1	2	96.67	0	3.33
PB	1–4	1	2	96.67	3.33	0
CIP	0.125–32	4	16	36.67	6.67	56.67
LEV	≤0.5–64	8	64	10	20	70
LOM	1− > 32	32	>32	3.33	0	96.67

*AMK, amikacin; GM, gentamicin; TOB, tobramycin; CZA, ceftazidime-avibactam; SCF, cefperazone-sulbactam; TZP, piperacillin-tazobactam; CAZ, ceftazidime; FEP, cefepime; ATM, aztreonam; IMP, imipenem; MEM, meropenem; CST, colistin; PB, polymyxin B; CIP, ciprofloxacin; LEV, levofloxacin; LOM, lomefloxacin.*

### Virulence Factors

**FIGURE 2:**
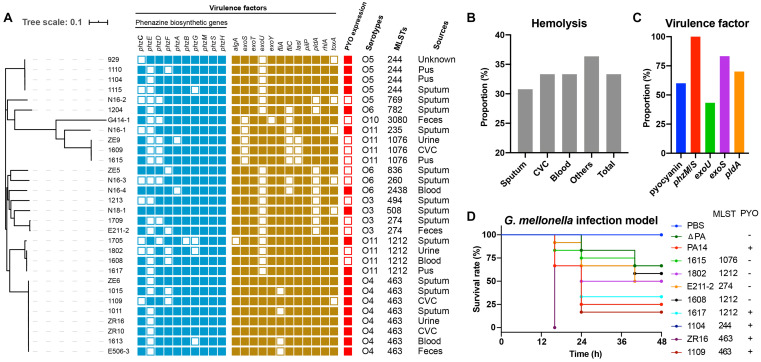
Assessment of the potential toxicity of *P. aeruginosa* isolates. **(A)** Phylogenetic tree and characteristics of all isolates. CVC, Central Venous Catheter; **(B)** Hemolysis proportion of different sampling sources. **(C)** The proportion of antimicrobial resistance-associated virulence factors, such as biosynthetic genes, *exoU*, *exoS*, *pldA*; and pyocyanin expression. **(D)** Larval survival rates in the *G. mellonella* infection model. Each isolate was challenged with 12 larvae for 48 h, with three biological replicates.

To determine the toxin-producing capacity of these isolates, we first carried out hemolysis tests, which showed that 33.33% of the isolates (10/30) displayed β-hemolysis (Figure 2B). Subsequently, we performed further characterization of the virulence in these isolates based on whole-genome sequencing analysis. The main virulence factors included mucoid related alginate (*algA*), T3SS effectors (*toxA*, *exoS*, *exoT*, *exoU*, and *exoY*), adherence factors (flagella, *fliC*, and *fliA*), quorum sensing gene (*lasI*), Type IV pili (*pil*P), rhamnolipid (*rhlA*), phenazine biosynthetic genes (*phzA-H*, *phzM*, and *phzS*), and the T6SS effector (*pldA*). All strains harbored *exoT*, *pilP*, *rhlA*, and *algA*, and 96.67% (29/30) of the strains carried *exoY*. Meanwhile, the prevalence rates of other factors, such as *lasI*, *toxA*, *exoS*, *fliC*, and *fliA* were 90, 80, 83.33, 73.33, and 86.67%, respectively (Figure 2A), suggesting that such genes are probably related to bacterial colonization and pathogenesis. Additionally, *exoU* and *pldA* were identified in 43.33% (13/30) and 70% (21/30) of the strains, respectively (Figure 2C). Intriguingly, all ST463 (O4) strains possessed exotoxin genes *exoU*, *exoS*, and *pldA*. Finally, 60% (18/30) of the strains produced pyocyanin *in vitro*, ranging from 0.02 to 3.2 μg/mL, based on triple quadrupole liquid chromatograph mass (LC/MS) analysis (Supplementary Figure 2 and Supplementary Table 2). Remarkably, all ST463 strains expressed high levels of pyocyanin (0.2–3.2 μg/mL), with an average of 1.48 μg/mL. Therefore, we deduced that ST463 (O4) might be a potential high-risk *P. aeruginosa* clone because of its high production of pyocyanin.

To explore whether there are any relationships between ST types and pyocyanin production in *P. aeruginosa*, we collected 27 genomes of pyocyanin-positive isolates from the *Pseudomonas* Genome Database (Supplementary Table 3.). We found that there was no common ST type that produced pyocyanin (Table 2). Taken together, these results suggested a positive connection between pyocyanin production and ST463-type *P. aeruginosa* strains.

**TABLE 2:** Information of pyocyanin-producing *P. aeruginosa* strains.

Strains	MLST	Source	Year	References
U018A	852	CF patient	2003	Cullen et al., 2015
2192	9	CF patient	2008	Cullen et al., 2015
39177	27	Cornea	2011	Cullen et al., 2015
Pr335	27	Hospital environment	1997	Cullen et al., 2015
M10	549	Surface water	2014	Cullen et al., 2015
AMT0023-30	1,394	CF patient	2010	Cullen et al., 2015
IST27	Unknown	CF patient	1996	Cullen et al., 2015
89	1,822	CF patient	2009	Caldwell et al., 2009
ID4365	560	Soil	2008	Rane et al., 2008
148	Unknown	Dolphin, gastric juice	2014	Grosso-Becerra et al., 2014
A5803	1,567	Pneumonia patient	2007	Cullen et al., 2015
LMG14084	316	Water	1960–1964	Cullen et al., 2015
39016	2,613	Cornea	2011	Cullen et al., 2015
40	2,238	CF patient	2009	Cullen et al., 2015
LES400	146	CF patient	2002	Cullen et al., 2015
LESB58	146	CF patient	1998	Cullen et al., 2015
M18	1,239	Rhizosphere	2005	Li et al., 2008
17	179	CF patient	2009	Caldwell et al., 2009
KK1	155	CF patient	2012	Cullen et al., 2015
57P31PA	274	COP patient	2009	Cullen et al., 2015
679	198	Non-CF patient, Urine	2011	Cullen et al., 2015
C3719	Unknown	CF patient	2000	Cullen et al., 2015
DK2	386	CF patient	1973	Cullen et al., 2015
TBCF10839	234	CF patient	2013	Cullen et al., 2015
1709-12	111	Non-CF patient	2004	Cullen et al., 2015
AMT0060-1	111	CF patient	2010	Cullen et al., 2015
AMT0060-3	111	CF patient	2010	Cullen et al., 2015

*MLST, multilocus sequence type; CF, cystic fibrosis; COP, chronic obstructive pulmonary; CVC, central venous catheter.*

To further evaluate the virulence potential of ST463, particularly the contribution of pyocyanin to survival rates, eight clinical isolates, 1615, 1802, E211-2, 1608, 1617, 1104, ZR16, and 1109 with different pyocyanin production levels, were used to challenge *G. mellonella* larvae. *P. aeruginosa* PA14, with high pyocyanin production and its mutant, *P. aeruginosa* ΔPA, with the deletion of pyocyanin producing *phz* genes (Zhu et al., 2019), were used as reference strains. We observed that the isolates that produced high levels of pyocyanin (*P. aeruginosa* 1617, 1104, ZR16, and 1109) induced higher mortality than those without pyocyanin production (*P. aeruginosa* 1615, 1802, E211-2, and 1608) (Figure 2D). This was in agreement with the finding that *P. aeruginosa* PA14 was more toxic to the larvae than *P. aeruginosa* ΔPA, indicating that pyocyanin plays an important role in the pathogenicity of *P. aeruginosa-*associated infections. Notably, the ST463 type isolates (*P. aeruginosa* ZR16 and 1109) exhibited higher virulence than *P. aeruginosa* PA14 and the other clinical isolates tested in this study. Altogether, these findings demonstrated that *P. aeruginosa* ST463, with high pyocyanin production, is a high-risk clone in patients, suggesting that more attention should be paid to the control of the dissemination of such clones in the clinic.

## Discussion

The increasing prevalence of chronic and hospital-acquired infections produced by MDR or extensively drug resistant (XDR) *P. aeruginosa* strains is associated with the increasing prevalence of transferable resistance determinants, particularly against carbapenemases and extended-spectrum β-lactamases (ESBLs) (Oliver et al., 2015). In this study, high proportions of antibiotic resistance (66.67–96.67% to the combinations of β-lactams, except CZA; 86.67–90% to carbapenems, 56.67–96.67% to fluoroquinolones, 66.67–80% to cephems, and 73.33% to monobactams) were mainly caused by the presence of multiple resistance genes. CZA is a novel approved combination in China in 2019, which is used to treat severe infections associated with CRPA. CZA is inactive against metallo-β-lactamases-producing stains, while is quite active against KPC-producing isolates (Horcajada et al., 2019). Our results confirmed that the *bla*_IMP_-positive isolate (N16-2) is the only CZA-resistant strain among the *P. aeruginosa* isolates tested. Given the extensive usage of CZA, CZA-resistant KPC-producing *Klebsiella pneumoniae* has been reported in different countries, caused by a point mutation of the *bla*_KPC–2_ gene (Gaibani et al., 2019; Hemarajata and Humphries, 2019). Therefore, it is crucial to identify the resistance mechanism before the regimens to improve antibiotic efficacy are considered. The widespread carbapenemases are metallo-β-lactamases of VIM- (Verona imipenemase) and IMP- (imipenemase) types in *P. aeruginosa* (Boulant et al., 2018). Notably, there were 16 KPC-strains (KPC-2) among the 18 carbapenemase-producing isolates in our study, which was consistent with our previous findings that *P. aeruginosa*, especially ST463, is a new carrier of *bla*_KPC–2_ surrounded by the MGEs IS*Kpn6*-like and IS*Kpn8* (Hu et al., 2015).

Epidemic outbreaks of *P. aeruginosa* MDR/XDR high-risk clones within hospital environments typically belong to ST111 (serotype O11), ST175 (serotype O4), and ST235 (serotype O12) (Horcajada et al., 2019). ST235 has been identified worldwide as being associated with *exoS*^–^/*exoU*^+^ (Maatallah et al., 2011) and the carbapenemases VIM, IMP, FIM, and NDM (Maatallah et al., 2011; Juan et al., 2017). Frequently, ST175 is observed to be a producer of VIM-2, whereas ST111 can produce KPC-2 carbapenemase (Oliver et al., 2015). Compared with these MDR/XDR isolates, ST244 is another large clonal complex frequently detected globally (Oliver et al., 2015; Horcajada et al., 2019). Our collection contained one ST235 and four ST244 isolates, while ST463 is the most prevalent clone associated with serotype O4. Although *exoS* and *exoU* are often mutually exclusive (Vareechon et al., 2017), we identified the coexistence of *exoS* and *exoU* in all eight ST463 strains. Together with the observation that all ST463 strains produce high levels of pyocyanin and cause high toxicity in infection models, these results suggested that clinical ST463 *P. aeruginosa* is probably a high-risk clone that might cause serious threats to human health because of its integrated MDR and virulence factors.

## Conclusion

The present study indicated that clinical *P. aeruginosa* poses a potential threat to human health because of the presence of multiple virulence factors and antibiotic resistance genes. The results suggested that the strain ST463 most likely emerged as a hypervirulent clone of *P. aeruginosa* as a result of a unique combination of pyocyanin production and virulence genes, including *exoU*^+^*/exoS*^+^, and *pldA*. Additionally, our study proves that the utility of genome sequencing in understanding and monitoring the epidemiology of clinically significant nosocomial clones, which will lead to improved control strategies. Nevertheless, the dissemination, evolution, and fitness cost of clone ST463 remain unclear.

## Data Availability Statement

Illumina sequences generated and used in this study are deposited and available at the NCBI website under BioProject ID: PRJNA716108 and part of PRJNA648026. All *P. aeruginosa* isolates (30) are available at https://www.ncbi.nlm.nih.gov/genome/. The specific BioSample accessions are listed in Supplementary Table 1. All other data generated or analyzed during this study are included in this article and it’s Supplementary File.

## Ethics Statement

The studies involving human participants were reviewed and Ethical approval was approved by the Ethics Committee of The Second Affiliated Hospital of Zhejiang University, School of Medicine (Number: 2020-319). Written informed consent for participation was not required for this study in accordance with the national legislation and the institutional requirements.

## Author Contributions

KZ: conceptualization. HL, QW, and HY: methodology. RZ, KZ, and BS: validation. YH and WP: formal analysis and writing-original draft preparation. YW: data curation. KZ and BS: writing-review and editing. YH, RZ, and ZK: funding acquisition. All authors have read and agreed to the published version of the manuscript.

## Conflict of Interest

The authors declare that the research was conducted in the absence of any commercial or financial relationships that could be construed as a potential conflict of interest.

## Footnotes

1 https://cge.cbs.dtu.dk/services/ResFinder/

2 https://cge.cbs.dtu.dk/services/MLST/

3 https://cge.cbs.dtu.dk/services/PAst-1.0/

4 http://www.pseudomonas.com
