# Supplementary material for: A Potential High-Risk Clone of Pseudomonas aeruginosa ST463
Source: Front Microbiol. 2021 May 28;12:670202. doi: 10.3389/fmicb.2021.670202 (PMC8193091; doi:10.3389/fmicb.2021.670202)
Supplement: Supplementary file 1 [file Data_Sheet_1.docx]

**Supplementary Table S1. The detailed information of the isolates.**

| **Isolates** | **MLST** | **Serotype** | **Patients** | | **Isolation Year** | **Sample Source** | **Ward** | **Carbapenemase gene** | **BioSample No.** |
| --- | --- | --- | --- | --- | --- | --- | --- | --- | --- |
|  |  |  | **Sex** | **Age** |  |  |  |  |  |
| 1011 | 463 | O4 | Male | 50 | 2010 | Sputum | Surgery ICU | *bla*_KPC-2_ | SAMN15616609 |
| 1015 | 463 | O4 | Male | 67 | 2010 | Sputum | Neuro ICU | NT | SAMN15616727 |
| 1104 | 244 | O5 | Male | 33 | 2011 | Pus | Burns | NT | SAMN15616734 |
| 1109 | 463 | O4 | Male | 52 | 2011 | CVC | Burns | *bla*_KPC-2_ | SAMN15616621 |
| 1110 | 244 | O5 | Male | 45 | 2011 | Pus | Burns | NT | SAMN15616640 |
| 1115 | 244 | O5 | Male | 58 | 2011 | Sputum | Burns | NT | SAMN15616641 |
| 1204 | 782 | O6 | Female | 80 | 2012 | Sputum | NICU | NT | SAMN15616730 |
| 1213 | 494 | O3 | Male | 83 | 2012 | Sputum | Neuro ICU | NT | SAMN15616732 |
| 1608 | 1212 | O11 | Male | 82 | 2016 | Blood | Neuro ICU | *bla*_KPC-2_ | SAMN15616661 |
| 1609 | 1076 | O11 | Male | 63 | 2016 | CVC | Neuro ICU | *bla*_KPC-2_ | SAMN15616662 |
| 1613 | 463 | O4 | Male | 37 | 2016 | Blood | Neuro ICU | *bla*_KPC-2_ | SAMN15616666 |
| 1615 | 1076 | O11 | Male | 45 | 2016 | Pus | Neuro ICU | *bla*_KPC-2_ | SAMN15616668 |
| 1617 | 1212 | O11 | Male | 24 | 2016 | Pus | Neuro ICU | *bla*_KPC-2_ | SAMN15616670 |
| 1705 | 1212 | O11 | Male | 74 | 2017 | Sputum | Neurology | *bla*_KPC-2_ | SAMN15616678 |
| 1709 | 274 | O3 | Male | 73 | 2017 | Sputum | Central ICU | *bla*_KPC-2_ | SAMN15616682 |
| 1802 | 1212 | O11 | Female | 88 | 2018 | Urine | Central ICU | *bla*_KPC-2_ | SAMN15616696 |
| N16-1 | 235 | O11 | Male | 84 | 2016 | Sputum | Central ICU | *bla*_GES-1_ | SAMN15616709 |
| N16-2 | 769 | O5 | Male | 79 | 2016 | Sputum | Respiratory Medicine | *bla*_IMP-25_ | SAMN15616710 |
| N16-3 | 260 | O6 | Male | 37 | 2016 | Sputum | Emergency ICU | NT | SAMN15616711 |
| N16-4 | 2438 | O6 | Female | 64 | 2016 | Blood | Respiratory Medicine | NT | SAMN15616712 |
| N18-1 | 508 | O3 | Male | 88 | 2018 | Sputum | Healthcare | NT | SAMN15616718 |
| ZE5 | 463 | O6 | Female | 55 | 2013 | Sputum | Neuro ICU | *bla*_KPC-2_ | SAMN15616640 |
| ZE6 | 463 | O4 | Male | 52 | 2013 | Sputum | Neuro ICU | NT | SAMN15616641 |
| ZE9 | 1076 | O11 | Female | 81 | 2013 | Urine | Neuro ICU | NT | SAMN15616647 |
| ZR10 | 463 | O4 | Male | 75 | 2015 | CVC | Neuro ICU | *bla*_KPC-2_ | SAMN15616647 |
| ZR16 | 463 | O4 | Female | 81 | 2015 | Urine | Neuro ICU | NT | SAMN15616649 |
| 929 | 244 | O5 | NA | NA | 2009 | Unknown | ICU | *bla*_KPC-2_ | SAMN18394805 |
| G414-1 | 3080 | O10 | NA | NA | 2018 | Feces | ICU | *bla*_KPC-2_ | SAMN18394806 |
| E211-2 | 274 | O3 | NA | NA | 2018 | Feces | ICU | *bla*_KPC-2_ | SAMN18394807 |
| E506-3 | 463 | O4 | NA | NA | 2018 | Feces | ICU | *bla*_KPC-2_ | SAMN18394808 |

**MLST, multilocus sequence type; NA, Not available; NT, Not detected; CVC, central venous catheter; ICU, intensive care unit; NICU, neonatal intensive care unit.**

**Supplementary Table S2. Mass spectrometry parameters of pyocyanin.**

| Precursor ion (m/z) | Retention time (min) | Product ion (m/z) | Q1 Pre Bias (v) | CE | Q1 Pre Bias (v) |
| --- | --- | --- | --- | --- | --- |
| 211.05 | 3.295 | 168.20^a^ | −11.0 | −35.0 | −29.0 |
|  |  | 183.20 | −11.0 | −24.0 | −20.0 |
|  |  | 196.10 | −10.0 | −26.0 | −21.0 |

**Supplementary Table S3. Accession numbers of 27 genomes from pyocyanin-product isolates in the Pseudomonas Genome Database**

| Strain | Assembly Accession | Strain | Assembly Accession | Strain | Assembly Accession |
| --- | --- | --- | --- | --- | --- |
| U018A | GCF_003698395.1 | 148 | GCF_000647595.2 | KK1 | GCF_003698585.1 |
| 2192 | GCF_000152545.1 | A5803 | GCF_003698605.1 | 57P31PA | GCF_003698025.1 |
| 39177 | GCF_003698545.1 | LMG14084 | GCF_003698745.1 | 679 | GCF_003698425.1 |
| Pr335 | GCF_003698795.1 | 39016 | GCF_000148745.1 | C3719 | GCF_000152525.1 |
| M10 | GCF_004123535.1 | 40 | GCF_900143995.1 | DK2 | GCF_000271365.1 |
| AMT0023-30 | GCF_003698455.1 | LES400 | GCF_000583935.1 | TBCF10839 | GCF_003698035.1 |
| IST27 | GCF_003698655.1 | LESB58 | GCF_000026645.1 | 1709-12 | GCF_001756495.1 |
| 89 | GCF_900144325.1 | M18 | GCF_000226155.1 | AMT0060-1 | GCF_003698325.1 |
| ID4365 | GCF_000647615.1 | 17 | GCF_004369995.1 | AMT0060-3 | GCF_003698635.1 |

**Supplementary Fig. S1. The biosynthetic process of phenazines**

The final phenazines were synthetized via four pathways (two for pyocyanin (PYO), one for 1-hydroxyphenazine (1-HP), and one for phenazine-1-carboxamide (PCN)) from chorismic acid, which was transferred by phosphoenolpyruvate. Phenazine-1-carboxylic acid (PCA) was converted via 2-amino-2-desoxyisochorismic acid (ADIC), trans-2,3- dihydro-3-hydroxyanthranilic acid (DHHA), 6-amino-5-oxocyclohex-2-ene-1- carboxylic acid (AOCHC), hexahydro‑phenazine‑1,6‑dicarboxylate (HHPDC), and tetrahydro‑phenazine‑1‑carboxylate (THPCA), successively by the products of the *phzE*, *phzD*, *phzF*, *phzA*/*B*, and *phz*G genes. Then, PCA was converted directly to PYO via 5-methylphenazinium phenazine 1-carboxylate (5MPCA), which could also be synthesized by 2-hydroxyphenazine (1-OH-PHZ). The other two results of PCA were PCN and 1-HP, respectively catalyzed by PhzH and PhzS. *phzA* or *phzB* were the core biosynthetic genes for all phenazines, and *phzM* and *phzS* were the core biosynthetic genes for PYO.

**Supplementary Fig.S2. Quantitative detection of PYO production in *P. aeruginosa*** **in vitro by LC/MS**

The extract was subjected to the LC/MS profile after dissolved in 90% acetonitrile. A) Base chromatograms and MS spectrum of pyocyanin. B) Pyocyanin level of *P. aeruginosa* isolates. The mass spectrum in the positive ion mode shows the precursor ion of m/z 211.05; the resulting fragment ions of m/z 196.10, m/z 183.20, and m/z 168.20 correspond to the loss of CH_3_, CO, and (CH_3_+CO) groups, respectively.

**Supplementary Fig.S3. The phylogenetic tree of core genome SNPs of 8 *P. aeruginosa* ST463 isolates**

Maximum likelihood phylogenetic tree built with core single nucleotide polymorphisms (SNPs) identified by mapping to the isolate 1011 reference genome, the number of SNPs is given in red text above the branches on which they were identified.
